# Are Nurse Coordinators Really Performing Coordination Pathway Activities? A Comparative Analysis of Case Studies in Oncology

**DOI:** 10.3390/healthcare11081090

**Published:** 2023-04-11

**Authors:** Maria-Ximena Acero, Etienne Minvielle, Mathias Waelli

**Affiliations:** 1 ARENES—UMR 6051, EHESP, French School of Public Health, University of Rennes, 15 Avenue du Professeur Léon Bernard, 35043 Rennes, France; 2 i3-CRG Ecole Polytechnique, CNRS, Institut Polytechnique de Paris, Route de Saclay, 91120 Palaiseau, France; 3 Gustave Roussy, 114 Rue Edouard Vaillant, 94800 Villejuif, France; 4 Global Health Institute, University of Geneva, 24 rue du Général-Dufour, 1211 Geneva, Switzerland

**Keywords:** nurse coordinator, activity, care coordination, non-coordination, activity of the patient pathway nurse coordinators in oncology

## Abstract

Patient Pathway Coordination (PPC) improves patient care quality and safety, particularly in oncology. PPC roles, such as nurse coordinators (NCs), have positively impacted the quality of patient care and reduced financial costs. However, NCs and their real activities in Health Care Organizations (HCOs) are unclear. Our aim was to identify, quantify, and compare all activities performed by NCs in oncology care settings from an organizational approach. **Methods**: We used qualitative and quantitative approaches based on case study principles. We accumulated 325 observation hours by shadowing and timing the activities of 14 NCs in four French HCO in oncology. Data analysis was conducted using an analytical framework to investigate the Activity of PAtient PAthway Nurse Coordinators in Oncology (APANCO). **Results**: Our research generated important findings: (1) NC roles and job titles are not standardized. (2) Non-coordination related activities are important in NC work content. Non-coordination times were consistent with distribution times between ward NCs and NCs in centralized structures. Ward NCs had higher non-coordination activities when compared with NCs in centralized structures. (3) PPC times varied for both ward NCs and NCs in centralized structures. Ward NCs performed less design coordination when compared with NCs in centralized structures, and this latter group also performed more external coordination than ward NCs. **Conclusions**: NCs do not just perform PPC activities. Their position in HCO structures, wards, or centralized structures, influence their work content. Centralized structures allow NCs to focus on their PPC roles. We also highlight different dimensions of NC work and training requirements. Our study could help managers and decision-makers develop PPC roles in oncology.

## 1. Introduction

Coordination is a key factor in improving patient pathways for chronic disease management [1], particularly in oncology [2]. Cancer patients are particularly affected by pathway fragmentation and coordination requirements, especially during the transition between ambulatory and inpatient care [2,3]. Failures in Patient Pathway Coordination (PPC) can lead to detrimental consequences in care quality (medical errors, poor remedial control of toxicity, and side effects from chemotherapy) [3,4], patient experiences [5,6], and unwarranted care spending [4].

Since the late 1980s, faced with an increased incidence of cancer and cancer survivors [7], and in response to coordination needs, new PPC roles were developed [8]. Primarily performed by nurses [9], some roles are now performed by former patients [10] or other demographic groups (social workers or physicians). These roles can be performed in a Health Care Organization (HCO) setting or in ambulatory care as part of disease management programs [8,11].

Different models have been proposed from a clinical perspective [12,13], defining the scope of practice for professionals seeking PPC roles in oncology. Similarly, the literature have described positive outcomes in terms of pathway quality [14,15,16] and financial savings [17,18] for these PPC nursing roles. However, as initiatives increase, little consensus exists on the real activity performed by these roles in an organizational environment.

**Figure 1:**
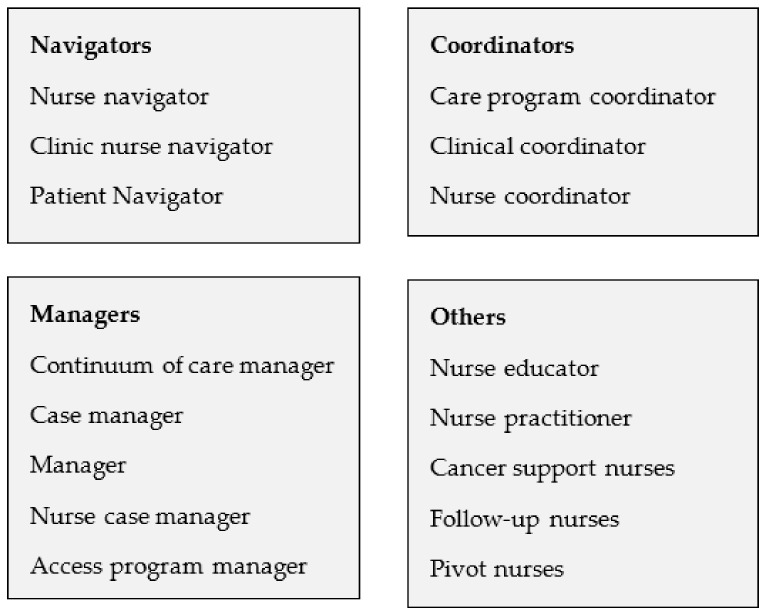
Diversity in nurse coordinator titles [19,20,21].

PPC nursing roles are often characterized by heterogeneous practices and functions, which are reflected in diverse job titles (Figure 1) [19,20,21], discrepancies in role definitions in HCOs across similar jurisdictions [19], overlaps between different roles [21], and limited training for coordination or navigation [13]. Given the multiplicity of job titles, we used the term nurse coordinator (NC) as a generic term for all jobs with a PPC nursing role.

Therefore, to advance our knowledge in this area and to capture NCs real activity in an organizational environment, NC activities should be investigated as they are performed in real-time work situations, i.e., for NCs in hospital contexts, their work content may be influenced by the organizational structure of the HCO [22].

Within this context, our aim was to identify and quantify all activities performed by NCs in oncology care settings. Rather than a clinical analysis, our goal was to propose an organizational analysis of NC activity [22]. Oncology is a good field to observe NCs; firstly, PPC requirements are numerous, especially during transitions between ambulatory and inpatient care [2,3]. Secondly, in several countries, government strategies have promoted NCs in oncology. In France, the 2009–2013 and 2014–2019 cancer plans funded NC programs in hospital and primary care settings [23].

Such analysis complements existing knowledge about the NC practice. In addition, at operational levels, such investigations can help managers and decision-makers develop PPC roles in oncology and other specialties.

## 2. Methods

A qualitative and quantitative approach were used based on comparative case study principles [24].

### 2.1. Field Selection

At four French HCOs, we identified NCs undertaking PPC roles for cancer patients.

HCOs were selected to include different forms of work organization (e.g., funding, size, type of coordination structure), a diversity of NCs and NC activities, and possible field access. HCO descriptions are shown in Table 1.

In terms of field access, we contacted the directors of the four HCOs and the professionals likely to be observed. We explained our study and obtained their consent to participate. We received no refusals.

### 2.2. Data Collection

The principal investigator (MXA) observed 14 NCs by shadowing [25] and timing activities based on time motion study principles [26]. To comprehensively investigate NCs at HCOs, MXA was present for 2–8 weeks. The observational work consisted of: (A) observing, describing, and timing NC activities during NC workdays; (B) identifying different actors who liaised with NCs and describing their relationship; and (C) collecting NC activity perceptions via informal exchanges. MXA maintained detailed notes on the content of activities and their objectives [24,25].

Exploratory interviews with NCs were conducted by MXA, proximity managers, and ward physicians to: (i) understand how NCs centralized structures and how wards functioned; (ii) identify which patients were being cared for, their position in the pathway, and for how long; and (iii) identify actor perceptions of NC relationships with care teams [24].

We also collected secondary sources such as reports, prescriptions, statistics, and job descriptions, which provided contextual information for our observations and accounted for contingency elements [24].

### 2.3. Data Analysis

Data analyses were performed using our previously developed Activity of PAtient PAthway Nurse Coordinators in Oncology (APANCO) framework [22]. Unlike other NC work models, APANCO not only accounts for NC activity but also highlights the influence of organizational strategies in an institutional context. APANCO allowed us to analyze the work content of NCs (Table 2).

Data analyses were conducted by MXA and discussed among the three researchers. Analyses were conducted in three steps:

1. Coding observations for NC: Activity Unit (AU) definition

To code observations, we defined Activity Units (AUs) as a collective action in a unique relational form with a shared purpose. The action could be performed between the NC and a professional or the NC and a patient (or their family). Its boundaries were determined by the extent of the NCs activity without interruption.

2. Individual analysis

After dividing NC observations into AUs, MXA coded information in Excel spreadsheets (one Excel sheet/category) using APANCO items as a grid. Standardizing coding analyses were performed in our preliminary research [22].

3. Comparing data between NCs

NCs had variable observation times; therefore, to compare observations between NCs, the AU time was divided by the total observation time. We quantified NC tasks as percentages, which provided a quantitative analysis of the volume of NC work. Considering the size of our sample for each HCO and the differences between them, we performed no other statistical analyses.

Interviews were complementary; they provided contextual data and perspectives on observational data. They were analyzed thematically using APANCO framework categories. Data/result interpretations were discussed during meetings between the study authors.

### 2.4. Research Ethics

According to the “Jarde Law”, the following types of research do not require an Institutional Review Board (IRB) [27,28]:

1. Research based on surveys and interviews with health professionals but not on the health of said professionals (e.g., burnout, addictions, etc.). In these cases, professionals are considered patients;
2. Research on teaching practices, particularly in health students, including simulations (as long as they do not involve the registration of any physiological parameters);
3. Research based on human and social science methods.

Our research was based on human and social science methods and involved health professionals. Nevertheless, we respected the five ethical principles (autonomy, justice, nonmaleficence, beneficence, and accountability). NCs were free to participate or withdraw from the study. To ensure participant anonymity, observations and interviews at sites were numbered consecutively using the NC acronym.

## 3. Results

The four HCOs were located in three French cities. We amassed 325 observation hours with 14 NCs. We also conducted 13 exploratory interviews with the following personnel: a gynecology department manager and three NCs (HCO-1): a coordination platform manager and three NCs (HCO-2); two NCs (HCO-3); and three NCs (HCO-4).

The majority of NCs were women (13/14), with an average age of 39 years. All 14 NCs who were shadowed had RN training. They also had prior experience in the oncology field before becoming NCs. Two NC career profiles were identified: before becoming NCs, ten individuals had care function roles in oncology wards (NCs 1, 2, 3, 4, 7, 8, 9, 10, 11, and 12) and four had department management roles (NCs 5, 6, 13, and 14).

### 3.1. Role and Job Title Heterogeneity

Roles were not standardized, and job titles differed from one HCO to another (Table 1). NCs worked in a single oncology specialty or across several specialties.

NCs had different roles in HCO work organizations. NCs 1, 5, 6, 7, 8, 13, and 14 belonged to inpatient or outpatient (e.g., day hospitalization) wards, while the reminder belonged to centralized structures (these structures are coordination structures outside inpatient wards, but they are part of HCO work organizations). NCs in centralized structures were organized by cancer type or treatment type.

In HCO-2, NCs 2, 3, and 4 belonged to a centralized structure that included a proximity manager, its own premises, and internal computer software. Each NC oversaw one or more oncology subspecialties, while the team covered all cancer types. In HCO-4, two centralized structures were apparent. In the first, NCs 9 and 10 followed-up patients on oral chemotherapy or targeted therapy with no patient distribution between NCs. The second structure coordinated transitions between hospital and ambulatory care; NCs 11 and 12 worked here, and patients were distributed according to cancer type. Both centralized structures shared premises, a secretary, and a proximity manager.

### 3.2. NC Non-Coordination Activities

In terms of work content, NCs also performed activities not related to their PPC roles. Depending on the context, the proportion of non-coordination activities varied from 9.3–47.8% of total NC work content. These activities involved clinical tasks, ward or staff management, and NC training or research projects.

NC career profiles influenced the type of non-coordination activity, e.g., NCs 5 and 6 at HCO-2 and NCs 13 and 14 at HCO-4, who previously held management positions in inpatient and outpatient wards, performed non-coordination activities related to ward management: scheduling, supply management, supervision, inpatient admissions, and bed management. NC1 at HCO-1, who previously had a role as a nurse anesthetist, performed significant clinical activity.

In other cases, non-coordination activities were linked to institutional demands. At HCO-3, the main non-coordination activities for NCs 7 and 8 involved regional NC training and participation in oncology PPC expert groups. For NC9 and NC10 at HCO-4, non-coordination activities were mostly related to NC training or research projects. For NC12 and NC11 at HCO-4 and NCs 2, 3, and 4 at HCO-2, non-coordination activity times were divided between institutional requests and walking from their premises to the wards to see patients.

**Figure 2:**
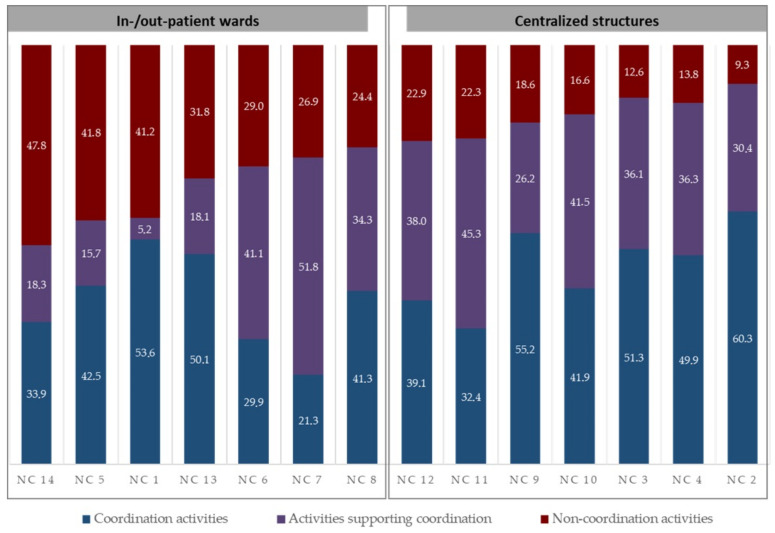
Comparative analysis of NC work content using the APANCO framework.

Strikingly, when we compared the proportion of non-coordination pathway activities between NCs, one factor was obvious: the distribution of non-coordination activities (percentages) corresponded perfectly with NCs working on inpatient and outpatient wards and those working in centralized structures (Figure 2).

We associated the proportion of non-coordination activity for each NC with the other variables presented in Table 1, but did not identify a connection.

### 3.3. Supporting Coordination Activities

The time dedicated to supporting coordination activities varied between 5.2% and 51.8% for NCs in inpatient and outpatient wards, and 26.2% and 45.3% for NCs in centralized structures (Table 1).

#### 3.3.1. Supporting NC Coordination Work

For NCs 1, 13, and 14 (inpatient and outpatient wards), and NCs 2, 3, 4, 9, and 10 (centralized structures), most of the time in this category involved activities supporting their own coordination work. For example, for NCs in centralized structures, preliminary work was performed before consultations or patient visits: NCs familiarized themselves with patient backgrounds, consulted medical reports and the latest check-ups, and prepared brochures or appointment schedules for patients. After patient consultations or professional discussions/meetings, NCs recorded these on internal centralized structure software and/or hospital software. Supporting NC coordination work represented 3.5–13.4% of NC activities in inpatient and outpatient wards and 19.5–28.7% of NC work in centralized structures.

#### 3.3.2. Supporting Physicians’ Coordination Work

NCs 6, 7, and 8 (inpatient and outpatient wards), and NCs 11 and 12 (centralized structures), supported physicians’ coordination work. For example, NCs pre-filled patient medical records before physician consultations, helped physicians fill in admission records, or pre-filled medical prescriptions for patients at discharge. These tasks represented 17.7–37.7% of NC activities in wards and 17–23.6% of NC activities in centralized structures.

### 3.4. PPC Activities: NCs in Inpatient and Outpatient Wards versus NCs in Centralized Structures

The proportion of time allocated to effective/true PPC was 21.3–53.6% for NCs in inpatient and outpatient wards and 32.4–60.3% for NCs in centralized structures (Table 3). These activities were performed with patients (or their families) or professionals. Four of the seven NCs in inpatient and outpatient wards (NCs 14, 5, 13, and 6) conducted coordination activities primarily with professionals. Five of the seven NCs in centralized structures (NCs 9, 10, 3, 4, and 2) mainly conducted coordination activities with patients.

#### 3.4.1. Centralized Structures Facilitate PPC Design or Patient Pathway Customization

In terms of patient pathway phases (design or implementation) where NCs were involved, differences were observed between NCs in inpatient and outpatient wards and centralized structures. The former group did not participate much in designing patient pathways (0–30.4% of coordination time; median: 2.2%), but they mostly implemented patient pathways. In contrast, NCs in centralized structures helped design or adapt pathways to meet patient needs (7.6–40% of coordination time; median: 20.4%). An example of such design coordination with other professionals (e.g., physicians) is shown in the following abridged field note:

Patient 21 is hospitalized at HCO-3 and is going to be discharged. The doctor prescribes him an intravenous medication three times a day to be administered at home. The NC suggests changing to a medication that can be given twice a day. The NC knows the patient lives in a village with limited access to home care nurses, so it will be difficult for a home care nurse to visit three times a day (NC3; field notes).

If only coordination activities with patients are considered for NCs 2, 3, and 4 (centralized structures), designing or adapting pathways is more important than implementation activities (51.1%, 52.8%, and 47.3% of coordination time with patients, respectively). Nursing support time is an example of a PPC design or adaptation activity. Nursing Support Time involves the comprehensive assessment of a patient’s health, condition, and needs (social and psychological). The NCs use this time to conduct therapeutic education and navigation. During consultations, which last approximately 45 min, NCs may propose domestic help or suggest a social worker, psychologist, or pain doctor. Importantly, at HCO-2, supportive care (dietician, social worker, pain physician, and psychologist) has increased by 15% since NC implementation (internal statistical data; HCO-2).

#### 3.4.2. Centralized Structures Facilitate External Coordination Activity

NCs in inpatient and outpatient wards primarily coordinated patient pathways in the hospital (external coordination; 11.7–35.3% of coordination time; median: 22.7%). In contrast, NCs in centralized structures had more external coordination activities (22.6–94% of coordination time; median: 71.6%), particularly for NCs in HCO-4 who coordinated ambulatory-hospital transitions (NCs 12 and 11) or provided follow-up at home for patients on oral chemotherapy (NCs 9 and 10).

## 4. Discussion

Our research generated important findings in two areas: NC work content and activity type distribution according to NC location.

### 4.1. NC Work Content: Time Dedicated to Coordination

Consistent with other studies [19,21], NCs performed, at different proportions, assessments, orientation, follow-up, and patient support. However, the proportion of NC activities dedicated to PPC varied considerably and did not represent the entire NC work content. Moreover, times ensuring smooth pathway transition between inpatient care and ambulatory care and pathways continuity outside the HCO (external coordination) were lower for some NCs. Our study suggested that many NCs had to address shortcomings or a lack of internal organization on HCO wards.

Allen [29] described factors (clinical or organizational) impacting on transitions between the sequence of care and external coordination; organizational factors include: (1) proximity and familiarity between wards or professionals, (2) the potential for boundary crossing to be supported by social interactions, (3) the degree of interpretative work demanded of practitioners in fabricating identities for the work purposes of others, and (4) the ease with which pertinent information was accessed [29]. Our study suggested that external coordination, despite barriers to its implementation, is indispensable as it provides continuity and fluidity in cancer patient pathways. In a systematic review on inter-professional and inter-organizational coordination, Karam et al. [30] recommended that NC roles should focus on external coordination to facilitate communications between different HCOs in patient pathways. Therefore, NCs must build and maintain links with professionals outside hospitals. Hanan-Jones et al. [31] described NCs roles as boundary spanners; their main function was to process external HCO information, which included gathering external information, filtering it based on relevance and priority, translating it into understandable terms for HCO members, and delivering it to relevant personnel [31,32].

PPC activities required time for preparation, verification, and downstream reporting. Some NCs supported the coordination activities of medical professionals (NCs 7 and 8 at HCO-3 and NCs 11 and 12 at HCO-4). Allen [33] reported that nurses chose to undertake or not work delegated by doctors in order to maintain patient care and treatment. Consistent with these findings, NCs at HCO-4 undertook work delegated by doctors and implemented “go faster” approaches for their patients. Nevertheless, NCs at HCO-3 on inpatient or outpatient wards perceived these tasks as acknowledgement or trust by physicians because they had delegated part of their activities. These differences were possibly explained by the fact that these NCs were performing relatively new jobs at this particular HCO and that funding was not permanent. These factors increased the need for professional acknowledgement, thus, sharing the work of physicians may have increased the NCs sense of legitimacy.

We previously showed [34] that professional perceptions and activity integration could be very different depending on ward organization. Similarly, our current findings also highlight important differences related to NC location.

### 4.2. Activity Differences According to NC Location

NCs in inpatient or outpatient wards spend more time on non-coordination activities (24.4–47.8%) when compared with NCs in centralized structures (9.3–22.9%), e.g., tasks related to ward management (12.9–26.9% for NCs 14, 5, 13, and 6). Similar findings were identified in 18 trauma networks in the UK [35]. NC administrative tasks included managing staff and ward rosters, ordering stock, answering telephones, attending meetings, and typing minutes. Spooner et al. [36] also highlighted that a portion of NCs work was dedicated to administrative tasks; the authors grouped tasks into a system support domain, which accounted for 19% of NC activity. Similar to NCs 14, 5, 13, and 6, NCs in the studies by Spooner [36] and Crouch [35] worked on inpatient and outpatient wards. The presence of NCs on wards may have influenced a higher proportion of administrative tasks in their daily work.

We suggest that NCs in centralized structures had an increased focus on PPC activities. These NCs spent more time designing or adapting pathways to meet the needs of patients compared to ward NCs. NCs in centralized structures also performed more external coordination activities when compared with NCs on inpatient and outpatient wards, whose coordination activities were mainly internal (65–88% of coordination time).

Nevertheless, such centralized structures in HCOs faced implementation issues related to historical work divisions. Indeed, division by ward is historically recognized in HCOs, which makes it difficult to implement centralized structures for mutualizing human resources in the HCO. Consequently, NCs working in centralized structures may receive little recognition, and their work may be poorly acknowledged by other ward professionals [19,37].

#### Study Limitations

Our study had some limitations. We developed an in-depth view of PPC activity content in HCOs and identified several organizational issues related to NC implementation. However, we had limited samples of both NCs and HCOs. Future, larger studies should be conducted to confirm our findings. We also focused on oncology, a field that has benefited considerably from many initiatives and significant PPC funding. Thus, to broaden the scope of our findings, further investigations must be conducted for other clinical conditions, e.g., NC roles in cardiology, and other organizational structures, e.g., NCs in ambulatory care.

## 5. Conclusions and Recommendations

Our results help us better understand NC work content and the boundaries affecting these activities. We showed that: (1) NC roles and job titles are not standardized, even within similar jurisdictions. (2) NCs do not only perform coordination activities, non-coordination activities are more important for NCs in inpatient and outpatient wards when compared with NCs in centralized structures. (3) NCs support the coordination work of other medical professionals. (4) NCs in centralized structures performed more PPC design/adaption activities and more external coordination activities when compared with NCs in inpatient and outpatient wards.

Our findings require us to consider the place of NCs in organizations. Our recommendations suggest to the managers that they promote centralized structures if the goal is to ensure a smooth transition between HCO and ambulatory care and also to promote actions to adapt care pathways to patient needs. Critically, we identified a risk of NCs straying from PPC activities; therefore, job descriptions must clearly identify tasks related to PPC.

Further studies should be undertaken to compare NC roles and their place in organizations in different countries. Similarly, these studies could be complemented by an organizational analysis of work and division of labor among professionals performing PPC roles.

Lastly, we highlighted different dimensions of NC activities and resultant training needs. To foster improved relationships between care team members, interdisciplinary training must be developed to deal with care coordination issues.

## Figures and Tables

**Table 1:** Health Care Organization (HCO) information.

	Not-Profit Hospital (HCO-1)	Public Academic Oncology Center (HCO-2)		Public Teaching Hospital (HCO-3)	Not-Profit Research Institute and International Cancer Center (HCO-4)		
**Information**							
**Beds**	140	59		898	365		
**Day-patient capacity**	26	49		147	94		
**Care days In-P ^†^**	8425	1843		120,667	19,807		
**Care days DH ^‡^**	6693	1753		-	7221		
**Average length of stay**	5.2	10.3		7.7	6.7		
**Link to**	Inpatient ward	Centralized structure	In- and Out- patient ward	Inpatient ward	Centralized structure	Centralized structure	Inpatient ward
**Hierarchical chain**	Gynecology ward nurse manager	Centralized Structure nurse manager	Ward nurse manager	Ward nurse manager	Centralized Structure nurse manager		Ward nurse manager
**Cancer type**	Breast cancer only	All types	All cancer types except hematology	All cancer types in the elderly. Respiratory system only	Patients on oral therapy	All types	All types
**Formalized since** **Role design**	Not yet formalized	2010	2017	2013	2015	2008	2016
	Professional (between the NC and the surgeon)	Institutional	Institutional	Professional design, which was institutionalized	Institutional		
**Human** **resources**	1 Coordination Support Nurse 3 Nurses Secretaries	5 Nurse Pivots 1 Pivot technical radiology assistant 1 Secretary 1 Advanced Practice Nurse	2 Liaison nurses 2 Nurse coordinators 2 Secretaries	2 Nurse coordinators 2 Scheduling nurses 2 Secretaries	2 Nurse coordinators 1 Research assistant 1 Secretary	5 Nurse coordinators 1 secretary 1 nurse’s aide	1 Nurse coordinator by ward 2 Secretaries
**Other** **resources**		- Coordination software (not connected to HCO software) - Premises		- Coordination software (unconnected to HCO software) - Patient assessment forms	- Internet portal - Scaling algorithms: Evaluate and rate event severity. - Orientation algorithms	- Coordination software (unconnected to HCO software)	- Inter-professional coordination forms
**Funding**	HCO	Government funding Associative funding HCO		Government funding	Government funding/HCO	HCO	HCO
**Study** **NCs**	NC1: Coordination Support Nurse	NC2: Pivot Nurse NC3: Pivot Nurse NC4: Pivot Nurse	NC5: Liaison nurse H ^†^ NC6: Nurse coordinator DH ^‡^	NC7: Nurse geriatric coordinator NC8: Nurse pneumology coordinator	NC9: Nurse coordinator NC10: Nurse coordinator	NC11: Nurse coordinator NC12: Nurse coordinator	NC13: Nurse medicine coordinator NC14: Nurse surgical coordinator

† In-P: Inpatient ward; ‡ DH: Outpatient ward (Day Hospitalization).

**Table 2:** APANCO framework categories.

Categories				Definitions
**Category 1**			**Coordination activity**	PPC is a collective activity between NCs, other professionals (medical, paramedical, or social), and cancer patients or their families. This collective activity involved sharing patient-related information (e.g., clinical, psychosocial, and service information) between pathway actors, regardless of their location (ambulatory/hospital), to ensure a smooth and continuous pathway.
*Sub- Categories*	1.1		*Design coordination activity*	coordination conducted during the development and updating of diagnostic and therapeutic strategies
			*Implementation coordination activity*	activities related to the diagnostic and therapeutic strategy, which depends on understanding work situations and defining corrective actions
	1.2		*Internal coordination activity*	coordination activities facilitating inpatient care
			*External coordination activity*	coordination activities ensuring continuity and smooth transitions between the hospital and primary care or outpatient care
	1.3		*Coordination activity between NCs and patients (families)*	coordination activities with patients, face-to-face or by telephone, or applications
			*Coordination activity between professionals and NCs*	coordination activity is conducted with healthcare professionals involved in the patient pathway, in person, by telephone, or through applications
	1.4		*Formal coordination activity*	coordination between professionals through standardized procedures
			*Informal coordination activity*	coordination between professionals through informal coordination mechanisms
**Category 2**			**Activities supporting** **coordination**	these activities do not directly influence patient pathway fluidity but are required for the smooth running of coordination actions. We categorized them into three types:
*Sub- Categories*		2.1	*Supporting NC coordination*	activities supporting NC coordination activities
		2.2	*Supporting other coordination*	activities supporting the coordination activities of other professionals
		2.3	*Relational coordination*	activities that promoted links and good understanding between the coordinator and other professionals
**Category 3**			**Non-coordination activities**	actions that do not coordinate the patient pathway. E.g.,
			Clinical activity, time visiting patients, training/expertise, ward management, and journey planning	

**Table 3:** Comparative analysis of NC activity in centralized structures versus NC activity in inpatient and outpatient wards. The APANCO framework was used to analyze NC work content.

		*Inpatient and Outpatient Wards*							*Centralized Structures*						
		HCO4	HCO2	HCO1	HCO4	HCO2	HCO3	HCO3	HCO4	HCO4	HCO4	HCO4	HCO2	HCO2	HCO2
		NC14 (%) *	NC5 (%)	NC1 (%)	NC13 (%)	NC6 (%)	NC7 (%)	NC8 (%)	NC12 (%)	NC11 (%)	NC9 (%)	NC10 (%)	NC3 (%)	NC4 (%)	NC2 (%)
***Category 1. Coordination***		**33.9**	**42.5**	**53.6**	**50.1**	**29.9**	**21.3**	**41.3**	**39.1**	**32.4**	**55.2**	**41.9**	**51.3**	**49.9**	**60.3**
*1.1*	*Design/*	0.40	1.0	0.0	1.6	0.0	6.5	10.3	3.0	3.8	7.9	8.5	20.5	15.4	20.1
	*Implementation*	33.6	41.6	53.6	48.6	29.9	14.8	30.9	36.1	28.6	47.3	33.3	30.8	34.6	40.2
*1.2*	*Internal/*	30.0	28.4	35.5	38.8	24.0	16.7	26.7	2.3	4.5	14.0	11.9	39.7	38.4	37.4
	*External*	4.0	14.1	18.1	11.4	5.9	4.5	14.5	36.7	27.8	41.2	30.0	11.6	11.6	22.9
*1.3*	*Professional/*	33.6	38.4	5.1	40.8	23.6	8.9	11.4	21.4	18.1	16.9	15.2	18.7	17.4	25.3
	*Patient*	0.4	4.2	48.4	9.3	6.3	12.4	29.9	17.6	14.2	38.3	26.6	32.6	32.5	35.0
*1.4*	*Formal/*	15.2	12.1	0.0	15.2	11.4	0.4	0.1	0.0	0.1	0.5	2.7	4.5	10.7	3.4
	*Informal*	18.4	26.3	5.1	25.6	12.3	8.5	11.2	21.4	18.0	16.4	12.5	14.2	6.7	22.0
***Category 2. Supporting coordination***		**18.3**	**15.7**	**5.2**	**18.1**	**41.1**	**51.8**	**34.3**	**38.0**	**45.3**	**26.2**	**41.5**	**36.1**	**36.3**	**30.4**
*2.1*	*Supporting NC coordination*	8.3	3.5	3.5	13.4	16.8	10.0	10.4	15.9	13.6	21.7	28.0	28.2	28.7	19.5
*2.2*	*Supporting other coordination*	5.6	2.6	0.2	2.8	17.7	37.7	18.8	17.0	23.6	1.6	9.7	1.1	0.6	5.3
*2.3*	*Relational coordination*	4.4	9.6	1.5	1.8	6.5	4.1	5.2	5.1	8.2	2.9	3.9	6.8	7.0	5.6
***Category 3. Non-coordination***		**47.8**	**41.8**	**41.2**	**31.8**	**29.0**	**26.9**	**24.4**	**22.9**	**22.3**	**18.6**	**16.6**	**12.6**	**13.8**	**9.3**
	*Clinical activity*	11.4	10.1	20.8	6.5	0.0	0.0	0.0	0.0	2.1	0.0	0.0	0.7	0.0	0.0
	*Time spent visiting patients*	0.1	1.6	6.7	4.4	0.0	0.0	2.8	5.7	2.7	2.4	0.6	4.2	3.1	8.1
	*Training/Expertise*	0.0	1.7	2.4	0.0	0.3	17.4	12.0	0.0	0.0	10.7	13.2	0.0	0.3	0.0
	*Ward management*	26.9	20.4	5.6	12.9	25.5	0.2	1.2	0.0	0.0	0.0	0.0	0.0	0.0	0.0
	*Journey planning*	2.1	0.3	5.6	8.0	1.5	1.6	3.4	1.5	1.9	2.9	2.5	2.5	3.7	0.0
	*Institutional requests*	7.3	7.6	0.0	0.0	1.6	7.7	5.0	15.7	15.5	2.5	0.3	5.3	6.7	1.2

* The percentages are calculated by dividing the category value (minutes) by the total time of NC observation and then multiplying the result by 100.

## Data Availability

Due to confidentiality, datasets are not publicly available.

## References

[1] Bodenheimer T. (2008). Coordinating Care—A Perilous Journey through the Health Care System. N. Engl. J. Med..

[2] Yatim F., Cristofalo P., Ferrua M., Girault A., Lacaze M., Di Palma M., Minvielle E. (2017). Analysis of nurse navigators’ activities for hospital discharge coordination: A mixed method study for the case of cancer patients. Support. Care Cancer.

[3] Gorin S.S., Haggstrom D., Han P.K.J., Fairfield K.M., Krebs P., Clauser S.B. (2017). Cancer Care Coordination: A Systematic Review and Meta-Analysis of over 30 Years of Empirical Studies. Ann. Behav. Med..

[4] Shrank W.H., Rogstad T.L., Parekh N. (2019). Waste in the US Health Care System: Estimated Costs and Potential for Savings. JAMA.

[5] Gardner K., Banfield M., McRae I., Gillespie J., Yen L. (2014). Improving coordination through information continuity: A framework for translational research. BMC Health Serv. Res..

[6] Levula A.V., Chung K.S.K., Young J., White K. (2013). Envisioning complexity in healthcare systems through social networks. Proceedings of the 2013 IEEE/ACM International Conference on Advances in Social Networks Analysis and Mining-ASONAM ’13.

[7] Mattiuzzi C., Lippi G. (2020). Cancer statistics: A comparison between World Health Organization (WHO) and Global Burden of Disease (GBD). Eur. J. Public Health.

[8] Chen A., Brown R., Archibald N., Aliotta S., Fox P.D. (2000). Best Practices in Coordinated Care.

[9] Dohan D., Schrag D. (2005). Using navigators to improve care of underserved patients: Current practices and approaches. Cancer.

[10] Freeman H.P. (2013). The History, Principles, and Future of Patient Navigation: Commentary. Semin. Oncol. Nurs..

[11] Ellrodt G., Cook D.J., Lee J., Cho M., Hunt D., Weingarten S. (1997). Evidence-Based Disease Management. JAMA.

[12] Baileys K., McMullen L., Lubejko B., Christensen D., Haylock P.J., Rose T., Sellers J., Srdanovic D. (2018). Nurse Navigator Core Competencies: An Update to Reflect the Evolution of the Role. Clin. J. Oncol. Nurs..

[13] Wells K.J., Battaglia T.A., Dudley D.J., Garcia R., Greene A., Calhoun E., Mandelblatt J.S., Paskett E.D., Raich P.C., The Patient Navigation Research Program (2008). Patient navigation: State of the art or is it science?. Cancer.

[14] Conway A., O’Donnell C., Yates P. (2019). The Effectiveness of the Nurse Care Coordinator Role on Patient-Reported and Health Service Outcomes: A Systematic Review. Eval. Health Prof..

[15] McMullen L. (2013). Oncology Nurse Navigators and the Continuum of Cancer Care. Semin. Oncol. Nurs..

[16] Byrne A., Hegney D., Harvey C., Baldwin A., Willis E., Heard D., Judd J., Palmer J., Brown J., Heritage B. (2020). Exploring the nurse navigator role: A thematic analysis. J. Nurs. Manag..

[17] Manderson B., Mcmurray J., Piraino E., Stolee P. (2012). Navigation roles support chronically ill older adults through healthcare transitions: A systematic review of the literature. Health Soc. Care Community.

[18] Wagner E.H., Ludman E.J., Bowles E.J.A., Penfold R., Reid R.J., Rutter C.M., Chubak J., McCorkle R. (2014). Nurse Navigators in Early Cancer Care: A Randomized, Controlled Trial. J. Clin. Oncol..

[19] Cantril C., Christensen D., Moore E. (2019). Standardizing Roles: Evaluating Oncology Nurse Navigator Clarity, Educational Prep-aration, and Scope of Work Within Two Healthcare Systems. Clin. J. Oncol. Nurs..

[20] Fillion L., Cook S., Veillette A.M., Aubin M., de Serres M., Rainville F., Fitch M., Doll R. (2012). Professional Navigation Framework: Elaboration and Validation in a Canadian Context. Oncol. Nurs. Forum.

[21] McMurray A., Cooper H. (2017). The nurse navigator: An evolving model of care. Collegian.

[22] Acero M.-X., Minvielle E., Waelli M. (2023). Understanding the activity of oncology nurse coordinators: An elaboration of a framework based on an abductive approach. Health Policy.

[23] Institut National du Cancer (2014). Plan Cancer 2014–2019: Guérir et Prévenir les Cancers: Donnons les Mêmes Chances à Tous, Partout en France, FÉVRIER.

[24] Tsoukas H. (1989). The Validity of Idiographic Research Explanations. Acad. Manag. Rev..

[25] McDonald S. (2005). Studying actions in context: A qualitative shadowing method for organizational research. Qual. Res..

[26] Lopetegui M., Yen P.Y., Lai A., Jeffries J., Embi P., Payne P. (2014). Time motion studies in healthcare: What are we talking about?. J. Biomed. Inform..

[27] Toulouse E., Masseguin C., Lafont B., McGurk G., Harbonn A., Roberts J.A., Granier S., Dupeyron A., Bazin J.E. (2018). French legal approach to clinical research. Anaesth. Crit. Care Pain Med..

[28] Salma I., Waelli M. (2022). Assessing the Integrative Framework for the Implementation of Change in Nursing Practice: Comparative Case Studies in French Hospitals. Healthcare.

[29] Allen D. (2014). Re-conceptualising holism in the contemporary nursing mandate: From individual to organisational relationships. Soc. Sci. Med..

[30] Karam M., Brault I., Van Durme T., Macq J. (2018). Comparing interprofessional and interorganizational collaboration in healthcare: A systematic review of the qualitative research. Int. J. Nurs. Stud..

[31] Hannan-Jones C.M., Mitchell G.K., Mutch A.J. (2021). The nurse navigator: Broker, boundary spanner and problem solver. Collegian.

[32] Aldrich H., Herker D. (1977). Boundary Spanning Roles and Organization Structure. Acad. Manag. Rev..

[33] Allen D. (2008). The nursing-medical boundary: A negotiated order?. Sociol. Health Illn..

[34] Michel L., Waelli M., Allen D., Minvielle E. (2017). The content and meaning of administrative work: A qualitative study of nursing practices. J. Adv. Nurs..

[35] Crouch R., McHale H., Palfrey R., Curtis K. (2015). The trauma nurse coordinator in England: A survey of demographics, roles and resources. Int. Emerg. Nurs..

[36] Spooner A.J., Booth N., Downer T.-R., Gordon L., Hudson A.P., Bradford N.K., O’Donnell C., Geary A., Henderson R., Franks C. (2019). Advanced practice profiles and work activities of nurse navigators: An early-stage evaluation. Collegian.

[37] Valaitis R.K., Carter N., Lam A., Nicholl J., Feather J., Cleghorn L. (2017). Implementation and maintenance of patient navigation programs linking primary care with community-based health and social services: A scoping literature review. BMC Health Serv. Res..

